# Effects of Dog-Based Animal-Assisted Interventions in Prison Population: A Systematic Review

**DOI:** 10.3390/ani10112129

**Published:** 2020-11-16

**Authors:** Beatriz Villafaina-Domínguez, Daniel Collado-Mateo, Eugenio Merellano-Navarro, Santos Villafaina

**Affiliations:** 1 Instituto de Mediación y Conciliación (IMECO), 46010 Valencia, Spain; bvillafainadominguez@gmail.com; 2 Centre for Sport Studies, Rey Juan Carlos University, 28943 Fuenlabrada, Spain; 3 Grupo de Investigacion EFISAL, Universidad Autónoma de Chile, Talca 3460000, Chile; emerellano@gmail.com; 4 Physical Activity and Quality of Life Research Group (AFYCAV), Faculty of Sport Sciences, University of Extremadura, 10004 Cáceres, Spain; svillafaina@unex.es

**Keywords:** inmates, prison, animal-assisted therapy, dog therapy, anxiety, stress, recidivism

## Abstract

**Simple Summary:**

Inmates usually suffer from depression, anxiety, or loss of empathy. Thus, animal-assisted therapies, and in concrete dog-based assisted therapy, were introduced in prisons in order to ameliorate the consequences of being into prison. Therefore, the aim of this study was to provide an up-to-date analysis of the research on the effects of dog-based animal-assisted therapy in the prison population. Results showed that mental health, emotional control, empathy, or academic skills could be improved after dog-based assisted therapy. Most of the studies included activities related to dog training, dog caring, or activities. The duration of dog-based therapies ranged between 60 and 120 min, with the frequency being between 1 and 3 days/week. In conclusion, these potential enhancements might lead to a reduction in recidivism and violence.

**Abstract:**

Background: Animal-assisted interventions, in concrete dog-assisted intervention, have been introduced in prisons to reduce recidivism as well as to improve the well-being of prisoners. Therefore, the aim of the present systematic review is to provide an up-to-date analysis of the research on the effects of dog-based animal-assisted therapy in prison population. Methods: An electronic search of the literature was performed, and 20 articles were included. The PRISMA guideline methodology was employed. Results: Included studies involved a total of 1577 participants. The vast majority of protocols included activities related with dog training, dog caring, or activities, which included vocational or educational components. Duration of dog-based therapies ranged between 60 and 120 min, with the frequency being between 1 and 3 days/week. Statistically significant improvements in prisoners were observed in 13 studies. Conclusions: Dog-based animal-assisted therapy may improve anxiety, stress, recidivism, and other social variables in male or female inmates.

## 1. Introduction

The proportion of both female and male inmates has been increasing since 2000. In this regard, the worldwide rate of female inmates has increased 50.2%, while the worldwide rate of male inmates grew 18.1%. Moreover, in the United States (EEUU), it is estimated that 221,600 young people were incarcerated [1] because of antisocial behaviors such as, theft, vandalism, bullying, or use of weapons [2]. People involved with criminal justice frequently are exposed to violence, traumatic experiences, or critical life events. Thus, the prevalence of posttraumatic stress disorder among sentenced prisoners is higher than that in the general population [3]. In this regard, prevalence of psychiatric disorders such as stress or anxiety is relatively high in prisons [4,5,6,7,8]. It is estimated that 10% of inmates experience depression or 50% experience antisocial personality disorder [7]. Due to the large number of people in prisons, which is more than 10 million individuals worldwide, and the large proportion of physical and mental health issues compared to the general population, there is a need to develop programs that help to reduce inmate prison infraction rates or violence and increase physical and mental health [9]. These intervention programs, which should be focused in psychosocial well-being, can be an effective way to maintain both staff and inmates’ safety within correctional facilities, while also reducing the likelihood of recidivism [10,11,12]. In this regard, animal-assisted interventions (AAI) are a promising strategy for these purposes, which is used in different countries [13,14,15].

The AAI is the umbrella term that refers to the deliberate and meaningful inclusion of animals into human health, wellbeing, or educational interventions [16]. Therefore, animal-assisted activities (AAA) and animal-assisted therapies (AAT) fall under this umbrella term. On the one hand, the AAA are less-formal interventions that aim to provide opportunities for motivational, educational, recreational, and/or therapeutic benefits to enhance quality of life but are not necessarily individualized or documented [17]. On the other hand, the AAT are defined as a goal oriented, planned, structured, and documented therapeutic intervention directed by health and human service providers in which an animal that meets specific criteria is an integral part of the treatment process [16,18]. Therefore, the interaction between the animal and the human could help to achieve a large variety of psychological and physical benefits for humans. These benefits could be explained by different reasons such as the biophilia hypothesis [19], which is explained as the genetically based propensity of humans to seek connections with other living organisms or an increase in oxytocin levels [20], which induces antidepressant and calming effects [21,22,23,24]. In addition, previous studies have shown a positive physiological response after being in contact with animals [25,26]. For instance, an increase in beta endorphins or dopamine production were found in humans [26]. Moreover, oxytocin levels seemed to increase [20]. This is relevant, since oxytocin is a peptide hormone and neuropeptide, released by the posterior pituitary, with antidepressant and calming effects [27]. This could be the reason why the AAI can reduce anxiety, depression [28], blood pressure [29], or cortisol [30], as well as increase wellbeing. However, methodological weaknesses in the AAI literature prevent a firm conclusion about the effectiveness of these approaches [31].

Different AAI approaches in terms of scope, aim, eligibility, or animal species involved have been described [32]. In this regard, dogs and horses are the most common therapy animal [18,33], but equine-assisted activities or therapies usually requires that the participant go to a specific facility where the horses are, thus the applicability in the prison context is reduced. The dog-based AAI consists of providing care and training for dogs, and sometimes, it includes vocational or educational components to enhance employability [15,34,35,36,37,38]. These kinds of interventions have shown positive effects on inmates. For instance, inmates who participated in dog-bases AAI needed less medication, were less violent to others, were less depressed, improved their social behaviors, and had fewer infractions at the same time that their self-esteem was increased and their stress levels were decreased [39]. In addition, since high dropout to behavioral intervention could be expected in this population, the inclusion of animals can increase adherence and motivation [40], which could be a key factor to consider, explaining the effectivity of AAI.

To our knowledge, one related review has been published in this area [41]. However, this review was focused in recidivism and included a total of 10 studies, with only three of them published in journals and including some unpublished material. Moreover, the search was conducted through March 2014 and authors did not follow the Preferred Reporting Items for Systematic reviews and Meta-Analyses (PRISMA) approach [42] to conduct the systematic review. Therefore, the aim of the present review was to provide an up-to-date analysis of the studies, published in scientific journals indexed in well-known databases, on the effects of dog-based AAI in female and male inmates, independently of their age (young or adults inmates), as well as to provide future directions.

## 2. Materials and Methods

The PRISMA guideline [42] has been followed to conduct the present systematic review.

### 2.1. Data Sources and Searches

Eligible literature articles were selected from PubMed, Web of Science (including Current contents connect, Derwent innovations index, Korean journal database, Medline, Russian science citation index, SciELO citation index), and Google Scholar databases. The search terms were: (a) the type of treatment (“dog” or “dogs” or “animal-assisted”) and (b) the population (“inmates” or “young criminal” or “juvenile offender” or “juvenile delinquents” or “prisoner” or “incarcerated” or “jail” or “prison” or “reform school” or “reformatory”).

The search was conducted by SV and checked by BVD. In case of disagreement, a consensus discussion directed by DCM was performed. The search ended on 31 March 2020.

The studies were included if they met the following inclusion criteria: (a) quantitative or qualitative randomized controlled trial or observational design, focused on dog’s assisted therapy (b) the target population were juvenile or adult delinquents. The following exclusion criteria were set: (a) the study was not written in English, French, Italian, Portuguese, or Spanish, (b) the article was presented as a summary at a conference or seminar, (c) it was a dissertation or thesis, (d) it was a professional experience.

### 2.2. Risk of Bias

The risk of bias of selected studies has been assessed by the Evidence Project risk-of-bias tool [43]. This tool includes eight items that are rated as yes, no, not applicable, or not reported: (1) cohort; (2) control or comparison group; (3) pre-post intervention data; (4) random assignment of participants to the intervention; (5) random selection of participants for assessment; (6) follow-up rate of 80% or more; (7) comparison groups equivalent on sociodemographics; and (8) comparison groups equivalent at baseline on outcome measures.

### 2.3. Data Extraction

Following the PRISMA methodology, the Participants, Intervention, Comparison, Outcome and Study design (PICOS) strategy was used [42]. The PICOS strategy allows us to extract these essential elements of the research question. Thus, this information was retrieved from the articles. In this regard, results reported the characteristics of the sample, as well as the interventions performed by the experimental and control group (when appropriate). Moreover, the details of the protocols, including durations of sessions, number of weeks, weekly frequency was extracted (when reported).

## 3. Results

### 3.1. Article Selection

**Figure 1:**
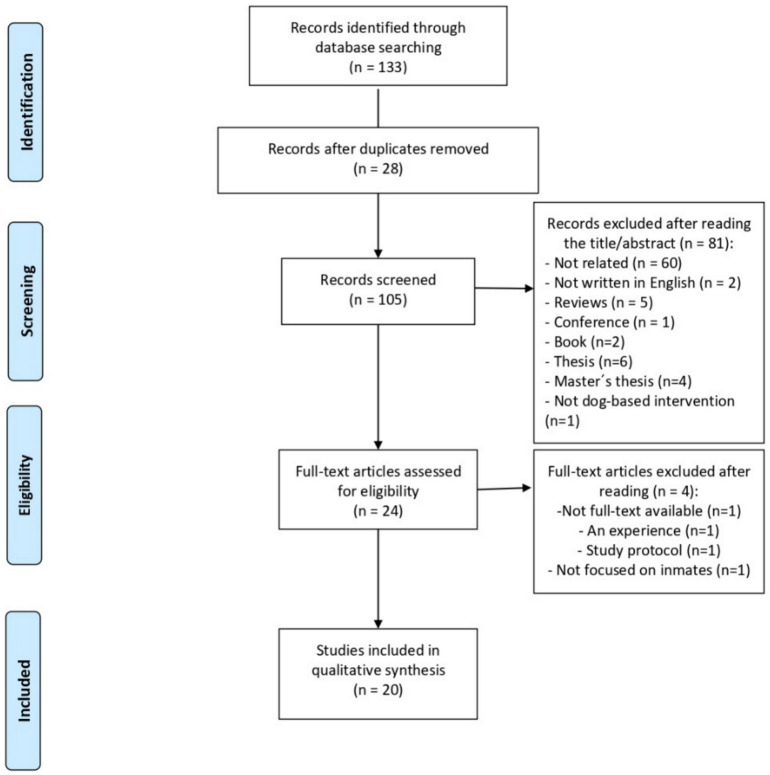
Flow chart for selection of studies.

Figure 1 shows the article selection process followed in this systematic review. A total of 133 articles were identified in the electronic databases: WOS (68 articles), PubMed (28 articles), and Google Scholar (37 articles). Twenty-eight articles were removed because they were duplicated. Moreover, 81 articles were removed after reading title/abstract (see Figure 1 for reasons). Of the remaining 24 articles, four were eliminated (see Figure 1 for reasons). After this exhaustive selection, twenty articles were included in the qualitative synthesis.

### 3.2. Risk of Bias

Risk-of-bias assessment was summarized in Table 1. The lack of control or comparison group (only 25% of the articles fulfilled this criteria) [32,44,45,46,47], random assignment of participants to intervention (15% of the articles fulfilled this criteria) [44,45,46], random selection of participants for assessment (0% of the articles fulfilled this criteria), or the follow-up rate (25% of the articles fulfilled this criteria) [39,45,46,47,48] are the most critical concerns.

### 3.3. Participants

Table 2 shows the population characteristics for each article. A total of 1295 participants are included in the present systematic review comprising ages between 13 and 69.

Seven studies (35% of the total) evaluated exclusively female inmates [14,44,49,50,51,52,53]. In this regard, four of them [14,44,49,53] were focused on psychiatric and emotional problems, mental health prison, multilevel security prison, and both adults and young female inmates.

Moreover, five articles (25% of the total) evaluated only male inmates [39,47,48,54,55]. Three of them [39,47,48] were focused on developmental disorders, psychiatric disorders, and drug-addicted inmates.

In addition, four articles [18,32,56,57] (20% of the total) evaluated both female and male inmates; one of them [18] was developed in a psychiatric prison. Additionally, four articles [45,46,58,59] (20% of the total) investigated young inmates.

### 3.4. Study Design

Table 2 displays the study design in each for the selected articles. A total of 14 (70% of the total) articles were classified as observational studies (an experimental group that was only assessed once at the end of the intervention) [14,18,39,44,48,49,52,53,54,55,56,57,58,59]. Of them, seven (50%) followed a quantitative [18,39,48,49,54,56,57] and seven (50%) a qualitative methodology [14,44,52,53,55,58,59].

Moreover, three articles (15% of the total) were classified as non-randomized controlled trials [32,47,51] (two groups with pre and post assessments but without randomization) and three (15% of the total) were classified as randomized controlled trials [45,46,50].

### 3.5. Intervention

Table 2 shows the intervention protocols in the treatment group for each article. In all the studies, the protocol includes a group of inmates who performed an AAI. Among the activities included in these programs were dog walking, dog training, taking care of dogs or rescue dogs as well as using the dog as emotional support during the therapy. Further details of intervention are summarized in Table 3.

### 3.6. Comparison Groups

Table 2 shows the control group protocols for each article. Fourteen of them [14,18,39,44,48,49,52,53,54,55,56,57,58,59] did not include a control group, while the design of remaining six studies [32,45,46,47,50,51] did. In this regard, treatment as usual or activities without dog presence were the most usual protocols for control groups. Only one article of those which present a control group included a passive control group (where participants did not participate in any therapy or activity) [32]. Further details can be checked in Table 2.

### 3.7. Outcomes

In order to summarize the outcomes, Table 4 (for quantitative studies) and Table 5 (for qualitative studies) were created.

Table 4 shows all the instruments and outcomes measures for the quantitative studies. Regarding the most studies variables in these studies, anxiety was measured in six articles [32,39,47,49,54,57], depression was measured in four of them [39,47,54,57], and, lastly, tension, irritation, vigor fatigue, distraction, and perspective taking were measured in two articles [32,39]. Three studies were classified as randomized controlled trials [45,46,50], two of them [45,50] showing within group improvements in social role, symptom distress, interpersonal relationships, or empathic concern, among others. Moreover, two articles were classified as non-randomized controlled trials [32,47] (two groups with pre and post assessments but without randomization), showing between groups effects on depression, sleep disorders, infraction rate, or state anxiety. The other seven articles [18,39,48,49,54,56,57] were observational studies, with reporting effects on recidivism, emotional state, tension, cortisol level, or emotional stability among other variables.

Furthermore, Table 5 shows the instruments and outcomes for the qualitative studies. One article was classified as a randomized controlled trial [51] and seven as observational studies [14,44,52,53,55,58,59]. Anxiety, depression, symbolism of the rescue dog, universal support, and psychological and emotional health were the most common outcomes among the qualitative studies. Other outcomes such as stress, self-stem, or empathy were also measured. The studies obtained the data using interview, report, observation, and survey questions (see Table 5 for further details).

## 4. Discussion

The aim of the present systematic review was to evaluate the effects of dog-based AAI in prisoners. Most of the included articles support the relevance and usefulness of this kind of therapy for male and female inmates. In this regard, many different variables could be improved after dog-based AAI in prisoners, including mental health variables, such as anxiety or depression, emotional outcomes, and other very relevant variables for prisoners, such as empathy, self-control, and even academic skills. The potential enhancements in those variables could also lead to a reduction in recidivism, which was also suggested in the previous review conducted by Cooke and Farrington [41]. However, although there are 20 articles included in this systematic review, given that some concerns may have increased the risk of bias, the interpretation of results must be done with caution.

The mechanisms that explain the improvements of these interventions are not usually investigated in AAI studies or in the analyzed studies. Furthermore, the complexity of the target population leads to a great heterogeneity of the studies and a poor methodological quality of many of them. This makes it difficult for this systematic review to draw conclusions in relation to the different mechanisms and specific strategies used in AAI.

A total of 1295 prisoners were included in this systematic review. In this regard, a similar number of men and women were included. The vast majority of the therapies were conducted in adults [14,18,32,39,47,48,49,50,51,52,53,54,55,56,57], in which usually both men and women equally benefited from the therapy. However, one intervention [57] reported evidence that dog-based AAI could be more affective among men compared with women. Authors hypothesized that this could be due to the observed better health status of males and also due to the lower stress factors compared to women. In this regard, it must be noted that female inmates often come from disadvantaged backgrounds and have poor education [57], and the frequency of physical and sexual abuse before going to prison is high [60]. Therefore, potential differences between male and female inmates may be relevant when designing AAI programs, but this hypothesis must be confirmed in future studies.

Regarding interventions duration and frequency, eleven articles [14,18,44,45,47,50,51,53,55,57,59] showed effects on relationships, psychological problems, empathic concern, emotional state, personal skills, and control of emotions. The proposed dog-based AAI in the vast majority of those studies had a duration of 60 min, one day a week. However, previous studies focused on psychological therapies indicated that the longer the intervention the greater the effects [61,62]. Thus, future studies should investigate the role of duration in the effectiveness of the AAI. In terms of the activities to be carried out, the most beneficial are related to keeping in touch with rescued dogs, dog training, and therapies focused on educational and interaction components. Moreover, future dog-based AAI should take into account some of the essential principles proposed by Samhsa [63]. In this regard, dog-based interventions should fulfil some criteria such as (1) safety; (2) trustworthiness and transparency; (3) peer support and mutual self-help; (4) collaboration and mutuality; (5) empowerment, voice, and choice; and (6) cultural, historical, and gender issues.

Regarding the design of the studies, only three of the twenty articles were randomized controlled trials. Therefore, results must be taken with caution, since the risk of bias and quality assessment showed critical issues in the vast majority of the articles. The lack of a comparison group, randomization, or the follow-up rate are the most critical concerns. Furthermore, the large heterogeneity of the people in prison in terms of sex, gender, age, educational background, mental disorders, drug addiction, etc., must be considered. Thus, larger randomized controlled trials are needed, and studies that follow animal-assisted intervention guidelines [64] are encouraged to enable the extraction of strong recommendations and conclusions. Nevertheless, the exceptionality of this research field, the nature of participants as well as situational and environmental context concerns make it almost impossible to achieve these research standards.

There are some limitations that should be considered. First, articles in English, French, Italian, Portuguese, or Spanish were included in the systematic review, so it is possible that studies in other languages were not found. Second, the quality of the articles (only three randomized controlled trials) and the heterogeneity of participants and outcomes mean that the conclusions of the systematic review must be taken with caution.

## 5. Conclusions

Dog-based AAI could be a useful tool to improve many different variables including mental health, emotional control, empathy, or academic skills in male and female inmates. However, the methodological quality of the included studies was not optimal, and the heterogeneity of the participants and outcomes was large. Thus, further studies with higher methodological quality are required and subgroups are encouraged to enable the extraction of strong recommendations and conclusions.

## Figures and Tables

**Table 1:** Risk of bias of randomized and non-randomized controlled trial using the Evidence Project risk-of-bias tool.

Study	Cohort	Control or Comparison Group	Pre/Post Intervention Data	Random Assignment of Participants to Intervention	Random Selection of Participants for Assessment	Follow-Up Rate of 80% or More	Comparison Groups Equivalent on Sociodemographics	Comparison Groups Equivalentat Baseline On Disclosure
Antonio (2017)	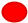	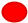	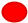	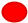	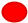	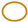	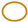	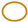
Collica-Cox (2018)	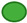	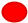	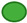	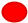	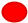	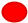	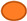	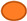
Contalbringo (2017)	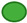	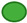	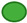	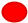	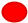	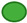	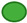	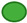
Cooke (2015)	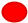	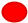	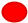	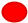	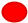	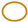	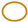	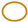
Dell (2019)	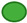	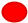	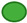	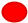	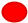	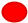	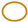	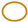
Flynn (2019)	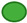	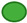	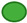	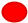	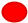	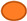	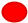	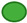
Holman (2020)	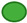	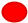	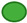	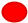	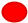	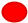	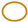	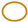
Jasperson (2010)	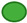	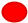	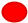	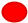	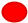	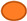	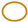	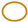
Jasperson (2015)	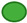	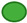	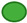	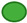	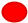	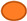	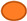	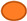
Koda (2015)	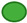	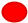	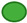	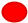	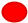	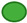	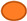	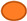
Koda (2016)	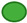	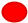	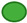	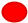	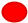	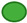	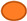	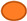
Kunz-Lomelin (2019)	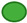	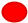	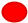	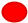	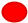	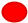	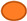	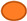
Leonardi (2017)	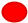	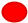	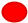	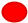	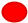	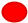	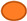	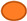
Minke (2017)	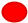	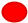	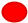	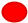	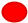	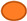	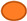	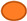
Minton (2015)	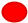	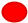	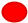	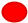	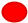	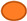	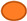	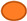
Seivert (2016)	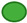	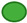	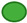	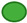	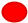	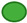	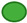	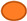
Smith (2019)	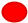	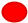	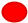	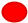	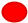	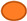	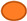	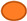
Smith and Smith (2019)	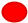	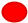	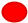	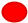	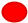	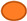	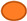	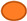
Stetina (2020)	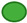	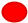	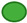	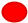	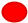	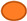	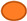	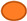
Syzmanski (2018)	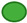	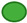	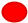	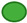	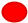	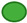	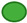	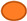

The Evidence Project risk-of-bias tool rated as: yes, no, not applicable, or not reported. Green: yes; red: no; orange: not reported; white: not applicable.

**Table 2:** Characteristics of the sample and the protocol.

Study/Year	Participants	Sample Size (N)	Age (SD)	Study Design	Control Group Protocol
**Antonio (2017)**	Female and male inmates and staff	62	45.36 (9.29) 43.85 (11.69)	Observational (Quantitative)	None
**Collica-Cox (2018)**	Female inmates	10	NR	Non-RCT (Qualitative)	Parenting, prison, and pups program without animal-assisted intervention
**Contalbringo (2017)**	Drug-addicted male inmates	22	EG: 35.5(13.83) CG: 42.9 (9.1)	Non-RCT (Quantitative)	Standard rehabilitation program
**Cooke (2015)**	Female inmates (Adults and young) with problems in psychological and emotional health	20 12 AI 8 YI	AI: 38.36 YI: 14–19	Observational (Qualitative)	None
**Dell (2019)**	Male and female inmates in psychiatric prison	3 1 F 2 M	48	Observational (Quantitative)	None
**Flynn (2019)**	Male and female inmates	229	EG: 39.4 (13.0) CG: 40.9 (11.0)	Non-RCT (Quantitative)	Passive control group. They did not participate in the program
**Holman (2020)**	Female inmates in mental health prison unit	6	31 (7)	Observational (Quantitative)	None
**Jasperson (2010)**	Female inmates with mental illness	5	26–42	Observational (Qualitative)	None
**Jasperson (2013)**	Female inmates	81	36	RCT (Quantitative)	Psycho-education and therapeutic intervention without dog
**Koda (2015)**	Male inmates with developmental disorders	72	26–60	Observational (Quantitative)	None
**Koda (2016)**	Male inmates with psychiatric or/and developmental disorders	73	26–60	Observational (Quantitative)	None
**Kunz-Lomelin (2019)**	Male inmates	17	19–58	Observational (Quantitative)	None
**Leonardi (2017)**	Young offenders	66	16–21	Observational (Qualitative)	None
**Minke (2017)**	Female inmates	12	39	Observational (Qualitative)	None
**Minton (2015)**	Female inmates (multi-level security prison)	30	50.23	Observational (Qualitative)	None
**Seivert (2016)**	Young inmates	117	15.7 (0.9)	RCT (Quantitative)	Animal education component and interaction component (Not engaged in dog training and were not assigned to any specific dog)
**Smith (2019)**	Male inmates (maximum-security prisons)	285	NR	Observational (Qualitative)	None
**Smith and Smith (2019)**	Young inmates (in adult prison)	31	21	Observational (Qualitative)	None
**Stetina (2020)**	Female and male inmates	81 50 M 31 F	29.3 (7.24)	Observational (Quantitative)	None
**Syzmanski (2018)**	Young inmates	73 43 EG 30 CG	NR	RCT (Quantitative)	Only walking dogs without teaching them

EG: experimental group; CG: control group; CG*: other control group; N: sample size; NR: not reported; RCT: Randomized controlled trial; SD: standard deviation; I: inmates; S: staff; AI: adult inmates; YI: young inmates; F: female; M: male; None: participants were not enrolled in any activity.

**Table 3:** Frequency, duration, and activities of the dog-based AAI interventions.

Study/Year	Intervention Duration (Weeks)	Session Duration (Minute)	Weekly Frequency (Days)	Activities Included in Session
**Antonio (2017)**	--	--	--	Dog training program.
**Collica-Cox (2019)**	8	120	2	The classes included Orientation and Parenting Styles; Effective Speaking; Effective Listening; Effective Problem Solving; Bonding Through Play and Reading; The Parent’s Job and The Child’s Job; Directions and Encouragement; Rules, Rewards and Consequences; Time Out with Back-Up Privilege Removal (non-violent discipline); Going Home To Your Children; Stress Management and Meditation; Healthy Adult Relationships; and CPR, First Aid, and AED certification for adults, children, and infants. The dogs will serve as emotional support during the class when difficult topics are discussed and the dogs will be incorporated into each lesson and serve as avatars/surrogates as women practice some of their skills. The therapy dogs will be available for the children and family members during the reunification/graduation day.
**Contalbringo et al. (2017)**	26	60	1	Experimental group is involved in dog-assisted therapy session, while control group is only part of standard rehabilitation program. Participants had to experience the interaction with the dog and they were involved in management and performance activities.
**Cooke (2015)**	8	--	--	Rehabilitation and educational program where participants have to train and care for shelter dogs.
**Dell (2019)**	26	30	--	Participants are part of animal-assisted therapy where it is intended to work human-animal bond.
**Flynn (2019)**	--	--	--	There are inmates who are part of dog training program, while there are a control group who are not part of dog training program.
**Holman (2020)**	8	30	1	Participants try to do clicker training exercise individually with the dog.
**Jasperson (2010)**	4 or 8	60	2 or 1	The treatment group implemented the use of a dog in order to facilitate the educational and therapeutic goals. In general, sessions were focused on the development of social skills, coping skills, and self-awareness. Each week, treatment group would sit in a circle on the floor and the dog would remain in the center of the circle. So, human–animal interaction was based on group member or animal initiative.
**Jasperson (2015)**	8	60	1	The group focused on personal safety, developing trust, being trustworthy, responsibility, understanding emotions, expressing emotions in a healthy manner, and learning new behaviors. Each week, group members sat in a circle on the floor and the dog remained in the center of the circle. Member or animal initiative prompted human–animal interaction.
**Koda (2015)**	12	70	1	The program was semi-structured with six themes, namely dog walking, dog obedience training, dog health check, dog massage, dog health care, and games with dogs. Each theme was repeated twice in successive weeks with different visitation dog–handler pairs.
**Koda (2016)**	12	70	1	The program was semi-structured and consisted of six activities, namely dog walking, dog obedience training, dog health check, dog massage, dog healthcare, and playing games with dogs. Each activity was repeated twice, in two successive weeks with different visiting dog–handler pairs.
**Kunz-Lomelin (2019)**	5	--	--	Participants are involved in a dog training course in which dogs receive Canine Good Citizenship.
**Leonardi (2017)**	8	--	3	Maximum 10 young men participating in each session. Participants learns how to train and care for the dogs, so they design training plans and use positive reinforcement methods to achieve their training goals.
**Minke (2017)**	--	--	--	Participants are involved in activities such as walking dog, cooking and dining, manufacturing key-hangers, and engaging in hobbies.
**Minton (2015)**	26–208	--	--	Dog training program in which women participated as dog trainers or assistants for the prison pup program.
**Seivert** **(2016)**	10	120	2	Sessions included a didactic 1 h animal education component and 1 h dog interaction component. The intervention group, in the interaction component, is involved in experiential learning in the form of positive dog training. While the control group do not engage in dog training and are not assigned to any specific dog.
**Smith (2019)**	--	--	--	Participants are involved in a dog-training program in which inmates are part of a program with rescue dogs.
**Smith and Smith (2019)**	--	--	--	Participants are involved in a dog-training program in which inmates are part of a program with rescue dogs.
**Stetina (2020)**	10	60	1	Dog-assisted group therapy is involved in competence and communication training that aims to enhance the social and emotional skills of the participants learning through interaction with the dog based on social and emotional skills that humans can learn from canines or socio-emotional interactions.
**Syzmanski (2018)**	10	120	2	Experimental group was learning to train dogs, while control group was walking the dogs. They also had classroom-based didactic sessions each week that focused on information about dog care, dog behavior, and humane treatment.

**Table 4:** Results synthesis of quantitative studies.

Authors	Instruments	Outcome Measure	EG Baseline	EG after Treatment	CG Baseline	CG after Treatment	Reported Effect
**Randomized Controlled Trials**							
Jasperson (2015)	Questionnaire (OQ)	-Social Role	14.33 (4.27)	12.65(3.80)	12.25 (4.39)	11.57 (4.82)	WG (EG)
		-Symptom distress	45.47 (11.77)	37.16 (13.98)	39.56 (14.31)	33.58 (13.06)	WG (EG)
		-Interpersonal relationships	21.40 (4.57)	19.91 (6.37)	19.82 (5.12)	17.98 (6.30)	WG (EG)
Seivert (2016)	TRFYSR	-Staff report internalizing	56.76 (9.09)	58.36 (9.50)	55.72 (9.18)	55.84 (9.62)	WG (EG/CG)
		-Youth report internalizing	55.43 (10.74)	56.33 (11.04)	53.89(11.09)	55.07 (10.86)	WG (EG/CG)
		-Empathic concern	17.74 (5.54)	17.67 (5.42)	16.85 (5.50)	18.43 (4.63)	WG (EG/CG)
		-Perspective taking	14.30 (5.92)	14.47 (5.58)	14.93 (5.12)	16.25 (5.35)	=
Syzmanski (2018)	Review and medical chart	-Future orientation	NR	6.13 (2.91)	NR	4.33 (3.15)	=
		-Cognitive growth	NR	8.05 (3.93)	NR	4.70 (2.52)	=
		-Self-awareness	NR	3.14 (1.27)	NR	3.04 (1.90)	=
		-Attachment	NR	6.79 (5.29)	NR	3.13 (3.10)	=
		-Attitude toward program	NR	3.12 (2.63)	NR	1.08 (0.95)	=
		-Positivity of emotion	NR	4.03 (1.21)	NR	2.97 (2.64)	=
**Non-Randomized Controlled Trial**							
Contalbringo (2017)	SCL-90-R	-Somatization	0.98 (0.89)	0.21 (0.24)	1.17 (1.30)	0.65 (0.74)	WG (EG)
		-Obsessive-compulsive symptoms	1.07 (0.61)	0.46 (0.29)	1.37 (1.05)	0.83 (0.53)	WG (EG)
		-Interpersonal sensitivity	0.60 (0.59)	0.23 (0.24)	0.70 (0.51)	0.52 (0.55)	=
		-Depression	1.34 (0.84)	0.45 (0.32)	1.10 (0.77)	0.83 (0.48)	WG (EG)/BG
		-Anxiety	1.39 (0.95)	0.44 (0.35)	1.07 (0.83)	0.73 (0.41)	WG (EG)
		-Hostility	0.57 (0.58)	0.43 (0.36)	0.67 (0.75)	0.53 (0.54)	=
		-Phobic anxiety	0.46 (0.55)	0.06 (0.07)	0.82 (1.40)	0.35 (0.51)	=/BG
		-Paranoid ideation	1.17 (0.72)	0.54 (0.49)	0.86 (0.74)	0.83 (0.59)	WG (EG)
		-Psychoticism	0.73 (0.62)	0.19 (0.16)	0.84 (0.77)	0.66 (0.41)	WG (EG)
		-Sleep disorders	1.78 (0.53)	0.63 (0.59)	1.89 (1.47)	1.00 (1.02)	WG(EG/CG)/BG
		-Global severity index	1.01 (0.54)	0.35 (0.19)	1.00 (0.82)	0.67 (0.43)	WG (EG)
Flynn (2019)	Survey	-Infraction rate	0.68	0.34	0.54	1.01	WG (EG)
		-Self-efficacy	NR	3.23 (0.47)	NR	3.15 (0.55)	=
		-State anxiety	NR	1.54 (0.48)	NR	1.70 (0.61)	BG
		-Trait anxiety	NR	1.91 (0.49)	NR	1.98 (0.56)	=
		-Empathy	NR	4.00 (0.75)	NR	3.81 (0.88)	=
		-Perspective taking	NR	3.42 (0.94)	NR	3.42 (0.93)	=
		-Fantasy	NR	3.09 (1.00)	NR	3.09 (0.96)	=
**Observational**							
Antonio (2017)	SAQ	-Reduced recidivism	NR	6.88 (2.36) M	NR	7.80 (2.37) F	BG
		-Non-violent incidents in prison	NR	3.76 (0.98) M	NR	4.09 (1.17) F	BG
		-Violent incidents in prison	NR	4.32 (0.79) M	NR	4.50 (0.72) F	=
		-Cooperative with correctional staff	NR	4.26 (0.73) M	NR	4.44 (0.90) F	=
		-Improved morale	NR	4.24 (0.82) M	NR	4.39 (0.91) F	=
		-Brings all inmates together as a community	NR	3.67 (0.98) M	NR	4.10 (0.86) F	BG
		-Provides inmates with marketable skills	NR	4.26 (0.89) M	NR	4.24 (0.88) F	=
		-Positive interactions with other inmates	NR	4.18 (0.75) M	NR	4.39 (0.61) F	=
Dell (2019)	Questionnaire	-Emotional state	3.3 (0.66)	4.8 (0.17)	NR	NR	WG (EG)
Holman (2020)	Generalized Anxiety Disorder 7-item scale (GAD-7)	-Levels of anxiety	16.16 (1.04)	4.33 (4.04)	NR	NR	=
Koda (2015)	Questionnaire(PGFSME)	-Tension	1.65 (1.10)	1.16 (1.20)	NR	NR	WG (EG)
		-Depression	1.46 (1.02)	1.14 (1.10)	NR	NR	=
		-Irritation	1.22 (1.01)	1.09 (1.13)	NR	NR	=
		-Vigor	1.91 (1.00)	2.12 (1.22)	NR	NR	=
		-Fatigue	1.35 (0.93)	1.25 (1.11)	NR	NR	=
		-Distraction	1.49 (0.90)	1.22 (1.11)	NR	NR	=
		-Anxiety	1.37 (1.04)	1.18 (1.16)	NR	NR	=
Koda (2016)	Monitoring salivary cortisol	-Cortisol level	Psychiatric disorders	NR	NR	NR	WG
			Development disorders	NR	NR	NR	=
			Psychiatric and development disorders	NR	NR	NR	=
Kunz-Lomelin (2019)	CES-D	-Depression	36.94 (11.62)	32.18 (12.50)	NR	NR	=
	GAD-7	-Anxiety	6.59 (5.81)	5.53 (5.94)	NR	NR	=
	RS-E	-Self esteem	17.76 (6.77)	17.41 (6.65)	NR	NR	=
	PCL-C	-PTSD	36.24 (14.42)	27.23 (10.49)	NR	NR	WG (EG)
	UCLA	-Loneliness scale	49.27 (8.63)	42.55 (14.34)	NR	NR	=
	RS	-Brief resiliency scale	3.82 (1.03)	4.10 (0.91)	NR	NR	=
Stetina (2020)	SEE	-Accept own emotion	22.30 (3.56) F	23.23 (2.69) F	21.46 (4.69) M	24.70 (3.8) M	WG (CG)/BG
		-Emotional flooding	21.93 (3.91) F	20.22 (3.81) F	21.36 (5.84) M	19.43 (5.03)	WG (ET/CG)
		-Lack of emotions	13.35 (2.81) F	13.29 (2.28) F	13.82 (3.97) M	12.20 (3.32) M	WG (CG)/BG
		-Somatic representation	24.77 (6.49) F	27.03 (4.78) F	25.20 (5.98) M	26.30 (5.45) M	=
		-Imaginative representation	17.90 (4.64) F	17.32 (4.93) F	16.40 (4.82) M	16.98 (4.68) M	=
		-Emotional regulation	12.16 (3.01) F	12.80 (2.7) F	13.30 (3.11) M	15.66 (2.37) M	WG (CG)/BG
		-Self-control	20.83 (3.66) F	21.74 (4.48) F	19.54 (4.36) M	22.64 (3.82) M	WG (CG)/BG
	EMI-B	-Anxious vs. free from fear	64.44 (9.48) F	65.11 (6.86) F	57.61 (12.97) M	49.16 (12.38) M	WG (CG)/BG
		-Depressive vs. happy	29.78 (4.37) F	28.22 (4.39) F	29.78 (8.04) M	22.00 (7.27) M	WG (CG)/BG
		-Tired vs. dynamic	29.56 (4.13) F	30.00 (2.03) F	29.00 (7.65) M	24.36 (6.84) M	WG (CG)/BG
		-Aggressive vs. calm	26.78 (5.01) F	29.22 (2.87) F	26.97 (7.71) M	22.63 (6.81) M	WG (CG)/BG
		-Inhibited vs. spontaneous	32.11 (5.67) F	32.44 (3.15) F	29.86 (6.24) M	27.08 (7.02) M	WG (CG)
		-Lonely vs. secure	30.67 (3.75) F	29.11 (2.37) F	30.50 (6.70) M	26.52 (4.99) M	WG (EG/CG)
		-Imbalanced feeling vs. well being	50.56 (11.12) F	49.33 (14.4) F	57.80 (14.5) M	45.36 (13.73) M	WG (CG)/BG
	SDQ-III	-Math	46.22 (10.86) F	44.32 (15.8) F	43.49 (17.45) M	47.79 (14.82) M	WG (CG)/BG
		-Verbal	43.45 (10.65) F	55.74 (10.85) F	53.67 (11.01) M	59.45 (10.79) M	WG EG/CG)/BG
		-Academic	46.29 (10.36) F	48.03 (12.48) F	45.79 (14.00) M	53.75 (15.19) M	WG (CG)/BG
		-Problem solving	48.64 (11.31) F	56.77 (7.12) F	51.93 (10.96) M	64.26 (8.76) M	WG (EG/CG)
		-Physical ability	44.48 (9.19) F	51.32 (11.73) F	51.83 (15.38) M	53.73 (14.34) M	WG (EG)/BG
		-Same sex peer relations	38.22 (7.48) F	52.54 (12.16) F	52.00 (10.77) M	58.97 (10.38) M	WG EG/CG)/BG
		-Opposite sex peer relations	41.03 (16.15) F	56.35 (7.03) F	53.42 (10.67) M	56.95 (11.39) M	WG EG/CG)/BG
		-Parent relation	39.19 (7.91) F	44.58 (15.47) F	46.97 (15.36) M	48.28 (16.08) M	=
		-Spiritual values/religion	57.22 (8.89) F	45.29 (12.54) F	49.81 (16.28) M	50.53 (20.33) M	WG (EG)/BG
		-Honesty/trustworthiness	49.61 (11.89) F	68.32 (12.06) F	66.4 (9.99) M	74.36 (8.14) M	WG EG/CG)/BG
		-Emotional stability	51.25 (13.67) F	50.19 (12.24) F	51.95 (12.64) M	63.36 (2.24) M	WG (CG)/BG
		-General esteem	54.19 (10.69) F	69.93 (11.41) F	66.08 (14.79) M	80.18 (10.71) M	WG (EG/CG)
		-Physical appearance	43.00 (11.01) F	41.45 (5.58) F	49.89 (11.84) M	51.53 (10.35) M	=

NR: not reported; BG: between groups; WG: within groups; EG: experimental group; CG: control group; F: female; M: male; SAQ: self-administered questionnaire; SCL-90-R: psychological test; PGFSME: Practical Group for Stress Management Education; RS-E: Rosenberg’s self-esteem; RS: Resiliency Scale; TRF: Teacher Report Form; YSR: Youth Self Report; SEE: Scales for Experiencing Emotions; EMI-B: Emotionality Inventory As a Measure of Well-Being; SDQ III: Self-Description Questionnaire III; PTSD: Post-Traumatic Stress Disorder; CES-D: scale that measures self-reported symptoms of depression); GAD-7: 7-item measure symptoms of anxiety; PCL-C: self-report to diagnose changes in PTSD; UCLA: loneliness scale.

**Table 5:** Results synthesis qualitative studies.

Authors	Instrument	Outcome Measures	EG Results	CG Results
**Non-Randomized Controlled Trial**				
**Collica-Cox (2018)**	Interview (DASS21)	Levels of stress	NR	−Stress, depression, and parental stress
		Anxiety		
		Depression		
		Self-esteem		+Self-esteem
**Observational**				
**Cooke (2015)**	Interview (Psychometric test)	Psychological and emotional Health	+Motherhood	NR
		Motherhood	+Transferable skills	NR
		Transferable skills	+Security	NR
		Security	+Trust	NR
		Trust	+Serving time	NR
		Serving time	+Social competence	NR
			+Interpersonal	NR
			dynamics	
**Jasperson (2010)**	Report GM and T’S	Anxiety	−Anxiety	NR
		Depressive symptoms	−Depressive symptoms	NR
		Self-awareness	+Self-awareness	NR
	Observation (MHP)	Social isolation	−Social isolation	NR
		Pro-social behaviors	+Prosocial behaviors	NR
**Leonardi (2017)**	Semi-structured interviews	−Dogs	+Educational engagement	NR
		−Positive effects	+Developing employability skills	NR
		−Motivation	+Enhancing well-being	NR
		−Charitable purpose		
		−Self-efficacy		
		−Improved skills		
		−Social impact		
		−Impulsivity		
		−Emotional management		
**Minke (2017)**	Interview	−Social relations	+The prison atmosphere and emotional support was better after treatment	NR
		−Emotional support	+Dog calm them and they defined prison as a “safe place”	NR
	Observation	−Normalizing the prison setting		
**Minton (2015)**	Semi-structured interviews	−Physical and emotional health	+Stress was reduced and losing weight	NR
		−Goal-directed	+Improve their self-concept, ability to reorganize	NR
		Behaviors	+Change their negative self-concept into a positive one	NR
		−Self-concept	+More empathic	NR
		−Empathy and self-control	+Ability to meet people	NR
		−Socialization		
**Smith (2019)**	Survey questions	−Symbolism of the rescue dog	+Dog represented unconditional love and care	NR
		−Universal support and spillover effects	+Dog as strengthening a sense of community in the unit	NR
		−Reinforcement of positive emotions	+Dog produced a stabilizing emotional effect	NR
		−Coping and linkage to the outside world	+Emotional stability	NR
		−Hope and transformation	+Positive emotions and coping	NR
		−Rotating dog handlers	+Participants need to rotate	NR
**Smith and Smith (2019)**	Survey questions	−Symbolism of the rescue dog	+Empathy and positive emotions	NR
		−Positive behaviors and rehabilitation	+Hope and rehabilitative developments	NR
		−A sense of normality	+Gave them a sense of normality and connection to the outside world	NR
		−Universal support	+Increase inmate’s positive viewpoint of dog	NR

EG: experimental group; CG: control group; GM: group member; T’S: therapist’s report; NR: non-reported; MHP: mental health professionals; DASS21: pre- and post-test data: inclusive of scales; “+”: increase; “−”: decrease.
